# Rethinking a Negative Event: The Affective Impact of Ruminative versus Imagery-Based Processing of Aversive Autobiographical Memories

**DOI:** 10.3389/fpsyt.2017.00082

**Published:** 2017-05-30

**Authors:** Christien Slofstra, Maarten C. Eisma, Emily A. Holmes, Claudi L. H. Bockting, Maaike H. Nauta

**Affiliations:** ^1^Department of Clinical Psychology and Experimental Psychopathology, University of Groningen, Groningen, Netherlands; ^2^Department of Clinical Neuroscience, Karolinska Institutet, Stockholm, Sweden; ^3^Department of Clinical Psychology, Utrecht University, Utrecht, Netherlands

**Keywords:** imagery, concrete, abstract verbal, rumination, autobiographical memory, processing mode, affect

## Abstract

**Introduction:**

Ruminative (abstract verbal) processing during recall of aversive autobiographical memories may serve to dampen their short-term affective impact. Experimental studies indeed demonstrate that verbal processing of non-autobiographical material and positive autobiographical memories evokes weaker affective responses than imagery-based processing. In the current study, we hypothesized that abstract verbal or concrete verbal processing of an aversive autobiographical memory would result in weaker affective responses than imagery-based processing.

**Methods:**

The affective impact of abstract verbal versus concrete verbal versus imagery-based processing during recall of an aversive autobiographical memory was investigated in a non-clinical sample (*n* = 99) using both an observational and an experimental design. Observationally, it was examined whether spontaneous use of processing modes (both state and trait measures) was associated with impact of aversive autobiographical memory recall on negative and positive affect. Experimentally, the causal relation between processing modes and affective impact was investigated by manipulating the processing mode during retrieval of the same aversive autobiographical memory.

**Results:**

Main findings were that higher levels of trait (but not state) measures of both ruminative and imagery-based processing and depressive symptomatology were positively correlated with higher levels of negative affective impact in the observational part of the study. In the experimental part, no main effect of processing modes on affective impact of autobiographical memories was found. However, a significant moderating effect of depressive symptomatology was found. Only for individuals with low levels of depressive symptomatology, concrete verbal (but not abstract verbal) processing of the aversive autobiographical memory did result in weaker affective responses, compared to imagery-based processing.

**Discussion:**

These results cast doubt on the hypothesis that ruminative processing of aversive autobiographical memories serves to avoid the negative emotions evoked by such memories. Furthermore, findings suggest that depressive symptomatology is associated with the spontaneous use and the affective impact of processing modes during recall of aversive autobiographical memories. Clinical studies are needed that examine the role of processing modes during aversive autobiographical memory recall in depression, including the potential effectiveness of targeting processing modes in therapy.

## Introduction

Autobiographical memories play a role in the onset and maintenance of major depressive disorder [hereafter also referred to as depression (1)]. Both the content of the autobiographical memories and the manner in which they are recalled may be disturbed in individuals with a diagnosis of depression, compared to never depressed individuals (1). Depression is associated with a tendency toward more negative and less positive memory recall (2, 3) and a more generic, less specific (overgeneral) nature of memory recall (4).

These memory disturbances in depression may be related to a more ruminative processing style during retrieval of memories. Rumination in depression can be defined as focusing on one’s depressive symptoms and the implications of those symptoms in a repetitive, passive, and abstract way (5). Rumination during autobiographical memory recall can be conceptualized as an abstract verbal thinking style revolving around “why” events occurred, and analyzing meanings, causes, and consequences of events (6). Rumination may thereby lead to the overrepresentation of abstract verbal material in the autobiographical knowledge base, disrupting the search process for a specific memory (i.e., memories of events that happened at a specific time and place), resulting in overgeneral memory recall [for reviews, see Ref. (4, 7)].

In depression, rumination may also be conceptualized as an emotion regulation strategy (8). Rumination, being an abstract and verbal thought process, resembles worry (5, 9). Following the avoidance theory of worry (9, 10), such reduced concreteness of thought may serve to avoid vivid recall of distressing images. Similarly, rumination is hypothesized to function to avoid extreme negative emotions associated with specific distressing images in autobiographical memory (11, 12). Ruminative (abstract verbal) processing has thus been proposed to “dampen” the negative affective impact of negative memories.

Another manner in which memories can be recalled is using situation-specific concrete processing, which concerns “how” the event occurred, which includes processing of the sensory experiences associated with the event (6). Such sensory processing is commonly referred to as mental imagery-based processing (13). In contrast to ruminative processing, imagery-based processing is proposed to enhance the impact of emotional material on negative and positive affect. In the short-term, concrete/imagery-based processing of aversive autobiographical memories is hypothesized to increase negative affect and decrease positive affect (14).

In the long term, however, rumination has been found to contribute to worsened mood and onset and maintenance of depression [for reviews, see Ref. (5, 6)]. Genetic vulnerability for rumination has been found to overlap with genetic vulnerability for depression (15) and, moreover, appears to mediate genetic vulnerability for depression (16). In contrast, more specific, imagery-based recall of aversive autobiographical memories may improve emotional processing of events and result in more functional recall of positive autobiographical memories. For example, in a non-clinical sample, concrete/imagery-based processing *during* exposure to analog trauma resulted in smaller negative affective impact at posttest and fewer intrusions in the following week than abstract/verbal processing (17). Thus, autobiographical memory disturbances in depression might also be interpreted as an imagery problem (18). In sum, recalling an aversive autobiographical memory in a more “ruminative” or in a more imagery-based manner may differentially influence its emotional impact and contribute to the onset and maintenance of depression.

The causal role of abstract/verbal versus concrete/imagery-based processing on the affective impact of negative stimuli has been demonstrated in various experimental laboratory studies. Non-clinical participants instructed to process negative, *non-autobiographical* stimuli in verbal, compared to an imagery-based manner experienced less affective impact (19, 20). Recently, the differential effects of abstract verbal and imagery-based processing during recall of *positive autobiographical memories* on positive affect were examined in two experiments with non-clinical samples (21). Processing mode was manipulated with instructions to recall the positive autobiographical memory in an abstract/verbal or in a concrete/imagery-based manner. As predicted, only concrete/imagery-based processing, and not abstract/verbal processing, resulted in increased positive affect in the first experiment. Interestingly, greater increases in positive affect were observed in individuals with higher levels of depressive symptomatology (although this increased positive affect was not maintained after a short delay). A second experiment demonstrated that a comparative verbal way of thinking (making comparisons between the memory and the current situation), rather than abstract verbal thinking, was detrimental to the positive affective impact of recall of positive autobiographical memories. This effect was again moderated by depressive symptomatology: the differential effect of verbal versus imagery-based processing was greater for individuals with higher levels of depressive symptomatology.

Differential effects of processing mode on affective impact have been replicated in a clinical sample. Werner-Seidler and Moulds (14) showed that, in both never-depressed and previously depressed individuals, experimentally induced abstract processing of positive autobiographical memories resulted in worse mood repair after a negative mood induction, compared to concrete processing of positive autobiographical memories. This effect could not be replicated using self-defining positive memories (22). Thus, experimental studies suggest that abstract verbal processing results in weaker affective impact of negative non-autobiographical stimuli and positive autobiographical memories than concrete, imagery-based processing. Therefore, adapting processing modes during recall of autobiographical memories may prove useful in clinical practice (1). Indeed, innovative interventions that target the processing rather than the content of autobiographical memories seem to be effective in reducing depressive symptomatology (23). However, this “affect dampening” property of ruminative processing compared to the “affect amplifying” role of imagery-based processing during recall of an *aversive autobiographical memory* has not yet been evaluated experimentally.

The current study investigated the differential effects of processing modes during recall of aversive autobiographical memories on affective impact. In this study, abstract verbal processing, defined as verbal processing of “why the event occurred,” was compared to imagery-based processing, defined as sensory-based processing of the specific event. Abstract versus concrete and verbal versus imagery-based processing may be distinct but also partially overlapping constructs. Manipulations that simultaneously alter the two dimensions may, therefore, complicate interpretation of the causal effects of abstract verbal versus imagery-based processing modes. This study, therefore, included a concrete verbal condition, defined as verbal processing of the specific event, defined as describing “how the event occurred” to investigate whether concrete verbal processing may have different effects on memory recall than abstract verbal or imagery-based processing.

The study included an observational and an experimental part. In the observational part, it was investigated how spontaneous use of the three processing modes (state measures of abstract verbal, concrete verbal, and imagery-based processing) and trait measures of rumination and imagery-based processing were associated with the impact of aversive autobiographical memory recall on negative and positive affect. In the experimental part, the causal effect of processing modes on affective impact was investigated by experimentally manipulating the processing mode (abstract verbal versus concrete verbal versus imagery) during retrieval of the same aversive autobiographical memory. How manipulations influenced affective impact during future recall of the memory was assessed at a follow-up assessment of the memory.

Finally, because previous research showed that depressive symptomatology may moderate the effect of processing modes on affective impact of memories (21), it was investigated whether depressive symptomatology interacted with processing modes on affective impact after aversive memory recall. Based on experimental studies with verbal versus imagery-based processing of negative stimuli (19, 20), it was hypothesized that verbal (concrete and abstract) processing of aversive autobiographical memories would result in weaker affective changes than imagery-based processing, both in positive and negative affect. Based on studies comparing abstract versus concrete processing (14, 21), it was hypothesized that especially abstract verbal processing would result in dampened affective impact relative to imagery-based processing.

## Materials and Methods

### Participants

A sample size of 33 per group (99 in total) was predetermined. Ninety-nine undergraduates (66 females) with a mean age of 21.4 (SD = 3.4) participated in exchange for remuneration. All students over the age of 18 could partake; there were no exclusion criteria. No data on ethnicity or socioeconomic status were collected. Typically, these samples include a majority of Caucasian students. Participants provided written informed consent and were fully debriefed afterward. Ethical approval was obtained from the University of Groningen Ethical Committee of the Psychology Department (13231-N).

### Measures

#### Affect Measures

To measure affect, participants completed the sentence “at this moment, I feel…” by rating eight affect adjectives on 0–100 visual analog scales running from “not at all,” to “entirely.” A wide range of emotions was included to capture the various emotional responses to aversive autobiographical memories (24). A negative affect (NA) scale was created by averaging the scores in response to the adjectives *nervous, anxious, sad, down, angry*, and *irritated*. The positive affect (PA) scale consisted of the average score of the adjectives *happy* and *content*.

#### State Processing Mode Measures

To assess self-reported state processing mode, three scales were construed (items in Supplemental Material).

*State abstract verbal processing* was assessed using a 3-item scale derived from the momentary ruminative self-focus inventory (MRSI; Mor, Marchetti, and Koster, unpublished manuscript).

*State concrete verbal processing* was operationalized as a 2-item scale, assessing to what degree participants thought in words and sentences [e.g., Ref. (20)].

A 4-item *state imagery-based processing* scale was based on assessment of the use of sensory channels, assessing to what degree during recall participants saw, heard, smelled, and felt the sensory properties of the memory. Because smell was reported by less than half of the participants, it was not included in the imagery measure. All items were presented on 0–100 visual analog scales (which ran from “not at all” to “completely”).

#### Trait Processing Mode Measures

Self-report measures of trait levels of depressive rumination and imagery-based processing were assessed.

*Trait rumination* was assessed using the brooding subscale, a short scale (5 items) to measure maladaptive rumination, of the Ruminative Response Scale [RRS; (25); Dutch version (26)]. The internal consistency of this subscale in the current sample was questionable (α = 0.66).

*Trait imagery-based processing* was assessed using the 12-item Spontaneous Use of Imagery Scale [SUIS; (27)]. This questionnaire was translated to Dutch and backtranslated to English and compared to the original by a bilingual expert. The internal consistency in the current sample was acceptable (α = 0.71).

#### Depressive Symptomatology

Depressive symptomatology was assessed using the Inventory of Depressive Symptomatology (IDS-SR; 28, 29). This 30-item self-report version of the questionnaire covers all diagnostic criteria for major depressive disorder as formulated in the DSM-IV (30). Participants indicated to what degree each symptom applied to them in the last 7 days on a 4-point scale (0–3). Total scores range from 0 to 84. The psychometric properties are acceptable (29), with a good internal consistency in the current sample (α = 0.84).

### Experimental Manipulations

Manipulation was performed using three instructions for each processing mode. The first instruction was presented at the start, the second after 20 s, and the third after 40 s of recall. These manipulations were derived from previous studies (20, 21, 31) and altered to differentially manipulate abstract verbal, concrete verbal and imagery-based processing while keeping all other aspects of the manipulations the same.

The instructions in the *abstract verbal condition* were to “think about why the event occurred,” “think about the deeper meaning of the event,” and “think about how this event has influenced you.”

In the *concrete verbal condition*, participants were instructed to “describe in your mind precisely what is happening in the memory,” “describe in your mind in what order the events occurred,” and “describe in your mind what each person in the memory said and did.”

The instructions in the *imagery condition* were “recall the memory with your mind’s eye. Try to recall the smells, sounds, and sensations as well,” “see what is happening in your memory,” and “see the colors and others details in the memory.”

### Selection of an Aversive Autobiographical Memory

Participants were instructed to select a specific autobiographical memory using the following instructions: “please recall a specific event that happened on a specific time and in a specific place that made you feel anxious or sad at the time and still has an emotional impact on you now.” The memory was briefly described to the experimenter who assessed whether the memory concerned a specific moment and a specific place. If not, participants were prompted to specify the memory or select another memory.

### Procedure

Participants were informed that the study investigated their ability to recall their autobiographical memories. After inclusion, participants were randomly allocated to one of three conditions (abstract verbal, concrete verbal, or imagery). Participants were seated behind a computer, on which the measures (questionnaires, affect measures, and processing mode measures) were presented throughout the study. Auditory instructions were given *via* headphones. Participants were instructed to ask the experimenter, who remained present, any questions that may arise during the experiment.

**Figure 1:**
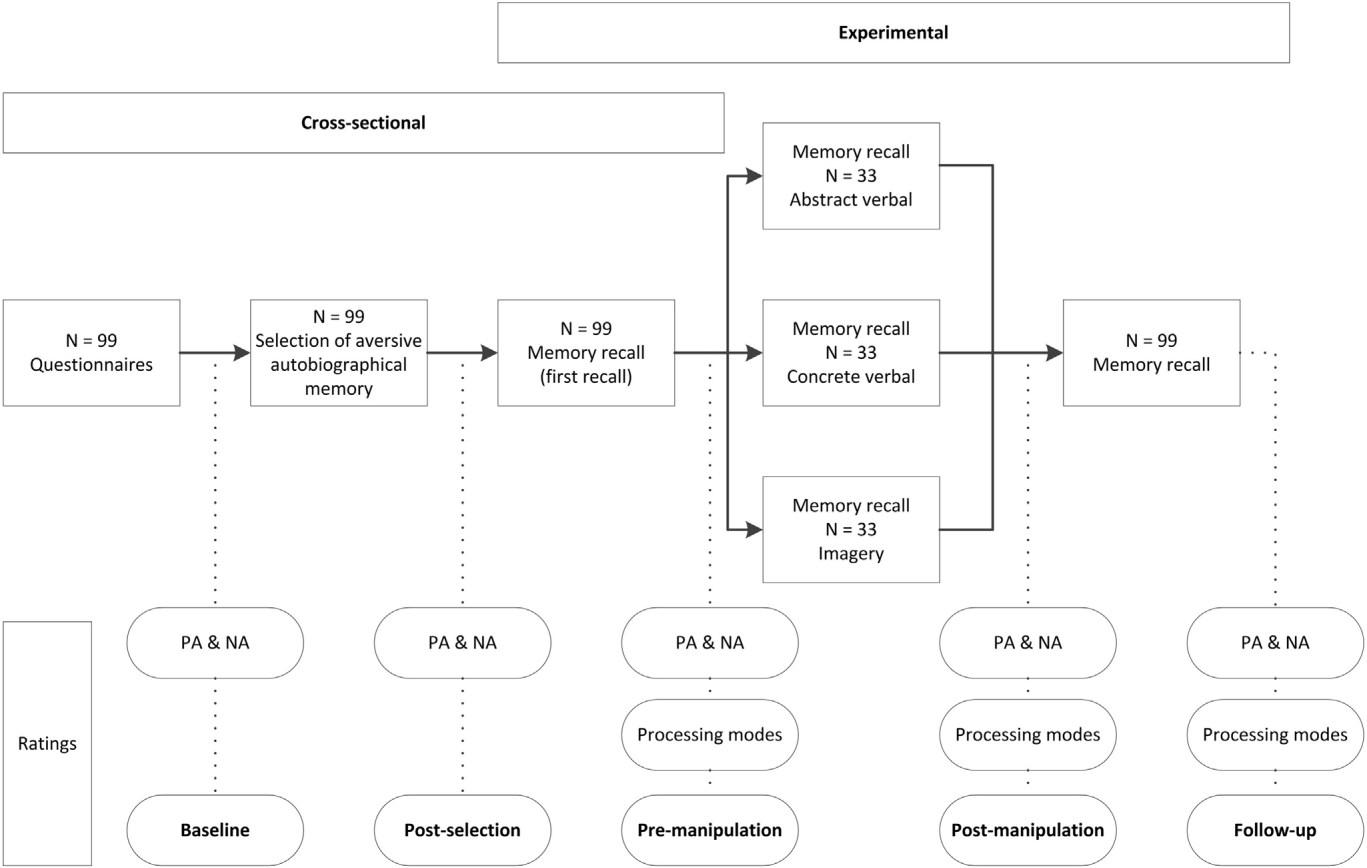
**Overview of study procedure of the observational and experimental part of the study**.

A schematic overview of the study procedure is depicted in Figure 1. All participants first filled out the questionnaires and then selected an aversive autobiographical memory. Affect was assessed before selection of the memory to establish baseline levels of NA and PA [baseline affect was assessed before selection of the aversive autobiographical memory, because we expected that memory selection may already impact negative and positive affect, which may already be under influence of processing styles. This expectation was confirmed by an additional post-selection assessment of affect after selection of the aversive autobiographical memory, showing that selection of the autobiographical memories resulted in a significant increase in NA [*t*(98) = 9.96, *p* < 0.01, *d* = 1.00] and decrease in PA [*t*(98) = −7.63, *p* < 0.01, *d* = −0.77].

Participants recalled their aversive memory three times during the experiment, for a pre-manipulation assessment (spontaneous processing during first recall), during the experimental phase (manipulated processing modes; post-manipulation assessment), and for the follow-up assessment (the effect of manipulating processing modes on affective impact during future recall). Each recall was immediately followed by ratings of the processing modes and affect measures. All participants were prompted to “please recall the memory as you usually recall it” for pre-manipulation and follow-up recall (both 24 s). During the experimental phase in between, participants received the manipulation instructions for abstract verbal, concrete verbal, or imagery-based processing, dependent on condition (see Experimental Manipulations) for 60 s.

Participants were free to choose whether they preferred to close their eyes during recall of memories or to keep their eyes open, but were instructed to remain consistent in doing so during recall throughout the experiment. The duration of the entire procedure was around 45 min per participant.

### Analyses

For the observational part of the study, affective impact of first recall was calculated as the change from baseline (before selection of the aversive autobiographical memory) to pre-manipulation (after first recall using spontaneous processing modes). It was analyzed how trait processing mode measures (RRS, SUIS) and state processing mode measures (VAS-scales; MRSI) were related to affect at baseline and the affective impact of first recall of the aversive autobiographical memory, using Pearson’s correlations.

The experimental part of the study had a mixed design. Change over time (within subjects) was compared between conditions (abstract verbal versus concrete verbal versus imagery-based; between subjects). To assess the causal effect of processing modes on affective impact of memories, hierarchical linear regression analyses were performed. In a first step, Condition [coded as a dummy variable for abstract verbal processing (1 for abstract verbal processing, 0 for other conditions) and a dummy variable for concrete verbal processing (1 for concrete verbal processing, 0 for other conditions)] was entered to predict changes in NA and PA from first recall to after manipulation (post-manipulation—pre-manipulation). In a second step, depressive symptomatology (group-mean centered) and the interactions between depressive symptomatology and conditions (dummy abstract and dummy concrete) were included in the model to assess whether depressive symptomatology moderated the effect of processing modes on affect. The latter analyses were repeated with changes in NA and PA from first recall to last recall (follow-up—pre-manipulation), to assess how manipulation of processing modes influenced future recall. All analyses were two-tailed.

## Results

### Relation between State and Trait Processing Mode Measures

The correlation coefficients are presented in Table 1. State abstract verbal (ruminative) processing during first recall was significantly and positively related to state imagery-based processing and trait rumination and imagery, although these correlations were modest. State imagery-based processing, in turn, was positively related to trait rumination and imagery. The trait measures of rumination and imagery-based processing were also significantly and positively related. In sum, state and trait measures of abstract verbal processing (or rumination) and imagery-based processing were positively related. In contrast, state concrete verbal processing was not significantly related to any of the other state or trait processing mode measures.

**Table 1:** **Correlations between state and trait processing mode measures, depressive symptomatology and affect at baseline, and change in affect between baseline and first recall (pre-manipulation)**.

		State			Trait		Depressive symptoms
		Abstract	Concrete	Imagery	Rumination	Imagery	
State	Abstract	–					0.17
	Concrete	0.11	–				0.13
	Imagery	0.23*	0.03	–			0.25*
Trait	Rumination	0.22*	0.03	0.23*	–		0.50**
	Imagery	0.27**	0.20	0.52**	0.36**	–	0.32**

Baseline	NA	0.22*	0.10	0.21*	0.33**	0.28**	0.63**
	PA	−0.19	−0.06	0.00	−0.10	−0.12	−0.49**
Change	NA	0.18	0.11	0.14	0.38**	0.23*	0.20*
	PA	−0.05	−0.05	0.06	−0.14	−0.04	0.04

*p < 0.05.

**p < 0.01.

*NA, negative affect; PA, positive affect; Concrete, concrete verbal, Abstract, abstract verbal, change represents first recall—baseline*.

### Observational: Relation between Processing Mode Measures, Affect, and Affective Impact of First Recall

Both state and trait measures of abstract verbal processing (or rumination) and imagery-based processing were found significantly related to higher levels of baseline NA. Trait measures of rumination and imagery-based processing were also related to change in NA after first recall of the aversive autobiographical memory. None of the state processing mode measures were significantly related to affective change after first recall (pre-manipulation). Thus, only trait measures of processing modes, and not state measures during recall, were associated with affective impact of the aversive autobiographical memory. Finally, higher levels of depressive symptomatology were related to higher levels of trait rumination and more state and trait imagery-based processing. Higher levels of depressive symptomatology were also related to baseline levels of affect (more NA, less PA), and greater negative affective change after recall.

### Manipulation of Processing Modes

The state processing-mode ratings per condition are presented in Table 2. There were no significant differences between conditions in pre-manipulation scores of affect or processing modes (all *p*’s > 0.05), demonstrating successful randomization. Examining whether the experimental manipulations resulted in relatively higher ratings of the condition-relevant processing mode during the manipulated recall demonstrated successful manipulation of processing modes. Repeated measures ANOVAs were conducted for the three manipulation tasks. The crucial effect was the interaction between time (pre-manipulation versus post-manipulation) and condition (abstract verbal versus concrete verbal versus imagery). As expected, for abstract verbal [*F*(2, 96) = 10.52, *p* < 0.001, $\eta_{\text{p}}^{2}=0.18$], concrete verbal [*F*(2, 96) = 12.72, *p* < 0.001, $\eta_{\text{p}}^{2}=0.21$], and imagery-based [*F*(2, 96) = 6.07, *p* < 0.01, $\eta_{\text{p}}^{2}=0.11$], processing, the changes in use of processing mode between conditions over time were significant.

**Table 2:** **Processing modes at pre-manipulation, post-manipulation, and follow-up per condition**.

	Abstract verbal		Concrete verbal		Imagery	

	*n* = 33		*n* = 33		*n* = 33	
Female, *n* (%)	19 (58%)		23 (70%)		24 (73%)	
Eyes open, *n* (%)	25 (76%)		28 (85%)		21 (64%)	

	**Mean**	**SD**	**Mean**	**SD**	**Mean**	**SD**

**Abstract verbal processing**						
Pre-manipulation	44.3	26.8	43.4	25.6	36.9	23.7
Post-manipulation	**58.9**	**27.5**	35.8	29.1	28.4	25.2
Follow-up	42.9	28.5	33.8	27.8	25.3	26.5
**Concrete verbal processing**						
Pre-manipulation	50.8	28.0	48.1	30.1	52.0	31.7
Post-manipulation	53.8	26.0	**68.7**	**27.5**	46.0	34.2
Follow-up	52.4	27.2	59.2	27.5	47.2	33.2
**Imagery-based processing**						
Pre-manipulation	50.8	22.9	56.3	23.5	56.8	21.3
Post-manipulation	40.5	24.3	53.3	25.1	**59.9**	**24.3**
Follow-up	39.9	23.1	49.5	22.8	48.9	20.2

*Condition-relevant processing mode ratings are in bold*.

*Analyses confirmed that all condition-relevant processing modes (in bold) had increased significantly (*p* < 0.01) from pre-manipulation to post-manipulation, relative to the non-relevant conditions*.

### Experimental: Effect of Manipulating Processing Modes on Affective Impact of Memories

The summaries of the models testing the causal effect of processing modes on change (from pre-manipulation to post-manipulation and pre-manipulation to follow-up) in NA and PA can be found in Table 3. It was tested whether manipulation of processing modes differentially influenced affective impact of aversive autobiographical memories in Model 1. No significant effects were found on NA or PA.

**Table 3:** **Regression model summaries testing the causal role of processing mode on affective impact of recall and depressive symptomatology as moderator**.

	Post-manipulation				Follow-up			
	NA change		PA change		NA change		PA change	
	B	β	B	β	B	β	B	β
Mean	0.4		−1.2		−0.1		−1.1	
**Model 1**								
Constant	2.06		−3.79		0.49		−3.33	
Abstract	−2.51	−0.12	3.70	0.12	−0.87	−0.04	3.56	0.11
Concrete	−2.35	−0.11	4.12	0.13	−0.76	−0.04	3.26	0.10
**Model 2**								
Constant	2.06		−3.79		0.49		−3.33	
Abstract	−2.51	−0.12	3.69	0.12	−0.87	−0.04	3.56	0.11
Concrete	−2.35	−0.11	4.12	0.13	−0.76	−0.04	3.26	0.10
IDS	−0.39*	−0.32*	0.41	0.22	−0.16	−0.13	0.19	0.10
IDSxAbstract	0.19	0.10	−0.17	−0.06	−0.27	−0.14	0.55	0.18
IDSxConcrete	0.63**	0.26**	−1.01**	−0.28**	0.55*	0.23*	−0.98**	−0.26**

*Abstract, abstract verbal condition dummy; concrete, concrete verbal condition dummy; imagery condition is reference category (constant)*.

*p < 0.10.

**p < 0.05.

*NA, negative affect; PA, positive affect, $R^{2}{}_{model1\;NA\;Post-manipulation}=\mathrm{0.01}$ (*p* = 0.51), $R^{2change}{}_{model2\;NA\;Post-manipulation}=\mathrm{0.06}$ (*p* = 0.14), $R^{2}{}_{model1\;PA\;Post-manipulation}=\mathrm{0.02}$ (*p* = 0.46), $R^{2change}{}_{model2\;PA\;Post-manipulation}=\mathrm{0.05}$ (*p* = 0.17). $R^{2}{}_{model1\;NA\;Follow-up}=\mathrm{0.002}$ (*p* = 0.93), $R^{2change}{}_{model2\;NA\;Follow-up}=\mathrm{0.08}$ (*p* = 0.049), $R^{2}{}_{model1\;PA\;Follow-up}=\mathrm{0.01}$ (*p* = 0.59), $R^{2change}{}_{model2\;PA\;Follow-up}=\mathrm{0.10}$ (*p* = 0.02)*.

Subsequently, it was tested whether depressive symptomatology moderated the effect of processing modes in Model 2. Adding depressive symptomatology as moderator resulted in a significant improvement of the model for NA ($R^{2}{}_{\text{change}}=0.08$, *p* = 0.049) and PA ($R^{2}{}_{\text{change}}=0.10$, *p* = 0.02) at follow-up. Depressive symptomatology moderated the differential effect of concrete verbal versus imagery-based processing for PA (*p* = 0.047). This interaction did not reach significance for NA (*p* = 0.08). These moderating effects in the concrete verbal condition were also significant at post-manipulation (*p*’s < 0.05), although they did not result in significant improvement of the model for NA ($R^{2}{}_{\text{change}}=0.06$, *p* = 0.14) or PA ($R^{2}{}_{\text{change}}=0.05$, *p* = 0.17).

**Figure 2:**
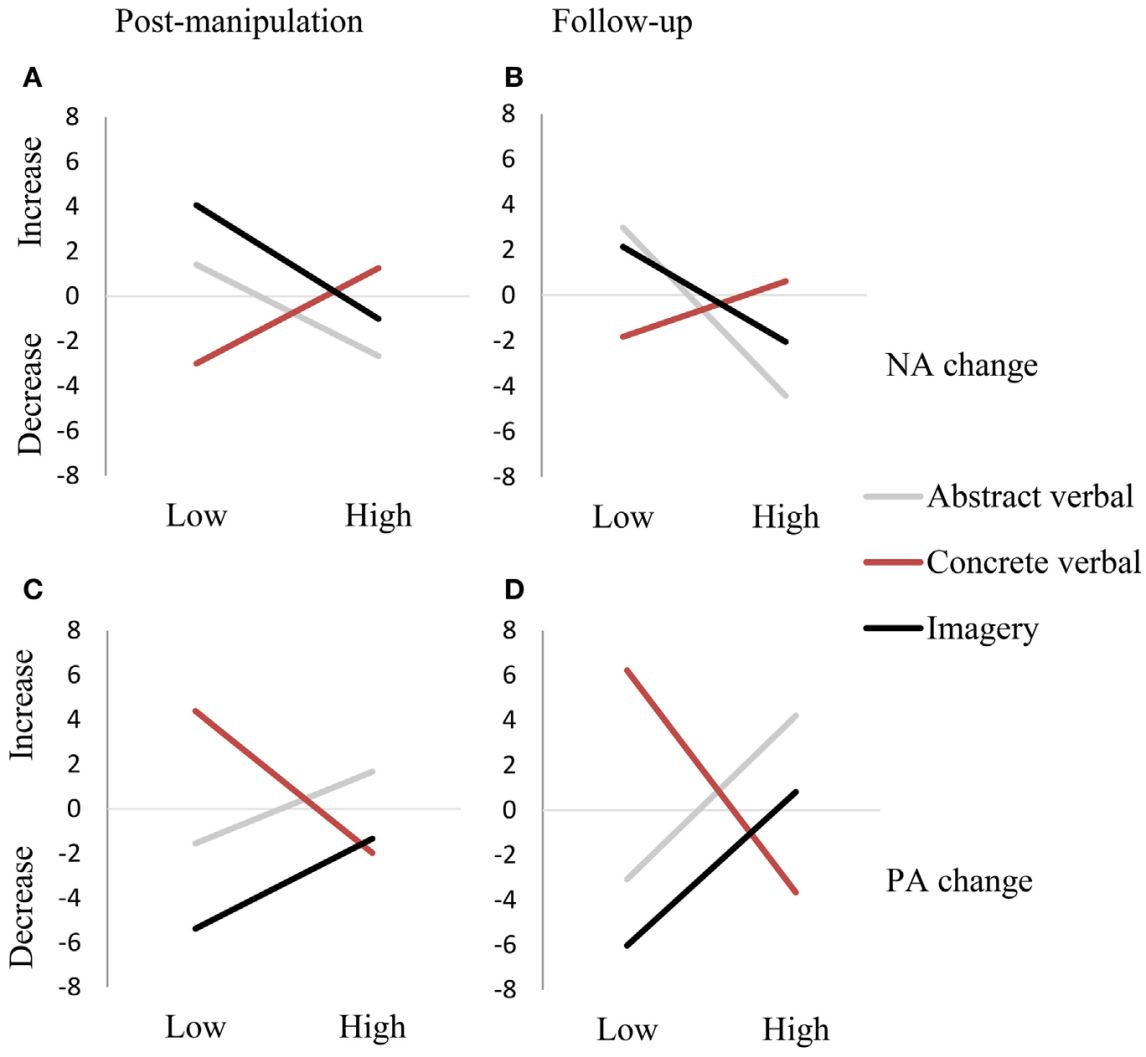
**Inspection of the three-way interaction (time × condition × depressive symptomatology), depicting change over time using a median split for depressive symptomatology for negative affective change at post-manipulation (A) and at follow-up (B) and for positive affective change at post-manipulation (C) and at follow-up (D)**.

The significant moderating effect of depressive symptomatology represents a three-way interaction between time (from pre-manipulation to post-manipulation and follow-up), condition (concrete verbal versus imagery), and depressive symptomatology. To unravel this three-way interaction, a median-split (median = 11) for depressive symptomatology was used. As can be seen in Figure 2, the differential effects of processing modes were more pronounced in the group with low depressive symptomatology. Here, as expected, concrete verbal processing resulted in weakened affective impact compared to imagery-based processing.

In sum, there were no main effects for condition or depressive symptomatology on affective change between pre-manipulation and post-manipulation or between pre-manipulation and follow-up. However, there was an interaction between depressive symptomatology and processing mode. Only in the low depressive symptomatology group did concrete verbal processing result in relatively weakened affective impact of memory recall.

## Discussion

The current study examined whether ruminative (abstract verbal) processing of aversive autobiographical memories dampens their affective impact. It was hypothesized that rumination may thereby serve to avoid distressing emotions for individuals with higher levels of depressive symptomatology, following avoidance theories of rumination [e.g., Ref. (11)]. The influence of processing modes on affective impact of an aversive autobiographical memory was investigated both observationally and experimentally. In the observational part of the study, higher levels of depressive symptomatology were associated with higher levels of trait rumination, as observed often before [for a review, see Ref. (5)]. However, depressive symptomatology was not significantly associated with ruminative (abstract verbal) processing during aversive autobiographical memory recall. Higher levels of trait (but not state) measures of both ruminative- and imagery-based processing were both correlated with higher levels of negative affective impact. In the experimental part, no main effect of processing modes on affective impact of autobiographical memories was found. A significant moderating effect of depressive symptomatology was detected. Only for individuals with low levels of depressive symptomatology, concrete verbal (but not abstract verbal) processing of the aversive autobiographical memory resulted in weaker affective responses, compared to imagery-based processing.

Thus, no “affect dampening” property of abstract verbal processing of aversive autobiographical memories was found. A potential explanation for this null finding is that heterogeneity in self-selected memories may have obscured significant main effects of processing modes. The role abstract verbal processing plays in affective impact of memory recall may be limited to the early stages of retrieving an autobiographical memory (7). Because all participants were instructed to select a specific memory, abstract verbal processing may not have truncated the retrieval process and, therefore, could not result in dampened affective impact. An alternative explanation could be that aversive autobiographical memories, as opposed to other negative material or positive autobiographical memories, are immune to the effect of processing modes [e.g., Ref. (32, 33)]. In other words, rumination might serve to avoid distressing emotions, for example when thinking about problems (34), but not during recall of aversive autobiographical memories. However, the fact that processing modes during recall do influence the affective impact of aversive autobiographical memories for people with low (but not high) levels of depressive symptomatology partly refutes this explanation. As such, the current results cast doubt on the hypothesis that rumination during recall of aversive autobiographical memories may serve to avoid the distressing emotions associated with the memory [e.g., Ref. (9–11)].

The effects of abstract verbal processing were contrasted to those of imagery-based processing. Observationally, higher levels of trait (but not state) imagery-based processing were associated with greater negative affective impact of aversive autobiographical memory recall. This finding is consistent with the hypothesized short-term “affect-amplifying” role of imagery-based processing (14). Importantly, no causal effects could be determined based on these observational findings. The experimental findings demonstrated that effects of imagery-based processing during recall of aversive autobiographical memories differ dependent on levels of depressive symptomatology. Only for individuals with low levels of depressive symptomatology, imagery-based processing amplified the positive and negative affective impact (after manipulation) and positive affective impact (at follow-up) compared to concrete verbal (but not abstract verbal) processing. Furthermore, higher levels of depressive symptomatology were (observationally) associated with more state and trait imagery-based processing and affective impact of aversive autobiographical memory recall.

So, when asked to retrieve a memory, persons with higher levels of depressive symptomatology tended to retrieve an aversive autobiographical memory in a more imagery-based manner and experienced greater increases in negative affect after retrieval of this aversive autobiographical memory. However, experimentally increasing their level of imagery-based processing during aversive autobiographical memory recall did not change the affective impact of recall for individuals with high levels of depressive symptomatology, whereas it did for individuals with low levels of depressive symptomatology. These results contrast with previous studies showing that low levels of depressive symptomatology were associated with more affective impact of positive autobiographical memory recall (35) and weaker effects of processing modes on this affective impact (21).

Taken together, these findings suggest that, in individuals with low levels of depressive symptomatology, spontaneous use of processing modes potentially contributes to affective benefit of positive autobiographical memories and dampens the negative affective impact of aversive autobiographical memories. In individuals with high levels of depressive symptomatology, inducing imagery-based processing may amplify affective impact of positive autobiographical memory recall (14, 21), but this does not appear to be the case for negative autobiographical memory recall. Depression may thus be associated with deficient imagery-based processing of positive, but not negative, material [for a review, see Ref. (18)]. The potential causal role of processing modes on affective impact of aversive autobiographical memory recall in (previously) depressed individuals remains to be examined further in future research. Based on the current findings, one may speculate that increasing imagery-based processing to increase positive affect in (previously) depressed individuals would at least not worsen the short-term affective impact of aversive autobiographical memories.

A concrete verbal condition was added to the experimental part of this study to disentangle the hypothesized abstract versus concrete dimension from the hypothesized verbal versus imagery dimension in processing style. Notably, concrete verbal processing was not significantly related to either abstract verbal processing (also on the verbal dimension) or imagery-based processing (also on the concrete dimension). Further, the correlational results suggest that trait ruminative and imagery-based processing are interrelated processes, positively associated with depressive symptomatology, more negative affect and greater negative affective impact of recall of aversive autobiographical memories. The relevance of distinguishing concrete verbal processing from imagery-based processing was further highlighted by the finding of differential effects of the concrete verbal versus the imagery condition for low depressed individuals. These results seem incongruent with the theory that abstract verbal processing, being less concrete, results in less imagery-based processing (9, 10), but rather point toward an “unemotional concrete verbal” processing style that seems unrelated to “emotional ruminative-imagery” processing. Future studies may further investigate the dimensions underlying cognitive processing of emotional material.

Some limitations should be noted. First, the time-course of this study was limited as post-measurement took place after manipulation without delay. In future studies, longer follow-up assessments of potential beneficial effects of concrete or imagery-based processing should be included, such as increased specificity of recall of positive memories (14). Second, the manipulation was moderate in strength. A stronger manipulation, possibly including an imagery practice task [e.g., Ref. (19)], would potentially have yielded more pronounced effects. Third, the effects in the experimental conditions can only be interpreted as relative to the other processing modes, because no neutral control condition was included in the design. Selection of a suitable neutral control condition in this design was complicated, given that distraction may also reduce rumination and negative affect (35, 36), and no instruction would not prevent individuals from further processing of the aversive memory in their habitual manner. Finally, the range of depressive symptomatology was restricted because the study was conducted in a non-clinical sample [which is common in this type of research, e.g., Ref. (19–21)]. Using a clinically depressed comparison group is recommended for future research, as it would have allowed for a comparison of levels and effects of processing modes between healthy and depressed individuals. The finding that the causal influence of processing modes on affective impact was only observed in individuals with low levels of depressive symptomatology highlights the need to examine how findings from non-clinical samples generalize to clinical samples of (previously) depressed individuals.

In sum, neither observationally nor experimentally were the current findings supportive of the avoidance hypothesis of ruminative processing during aversive autobiographical memory recall. The observational findings suggested that higher levels of depressive symptomatology are associated with higher levels of imagery-based processing and more affective impact of aversive autobiographical memory recall. The experimental findings suggested that affect-amplifying effects of imagery-based processing of aversive memories may be weakened in individuals with higher levels of depressive symptomatology. Ruminative (abstract verbal) and imagery-based processing appear interrelated phenomena, which are intertwined with depressive symptomatology and affect. Further studies in clinical samples are needed to gain understanding of the role of processing modes during recall of aversive autobiographical memories in depression, and how targeting processing modes in therapy may influence affective impact and depressive symptomatology.

## Ethics Statement

This study was carried out in accordance with the recommendations of University of Groningen Ethical Committee of the Psychology Department with written informed consent from all subjects. All subjects gave written informed consent in accordance with the Declaration of Helsinki. The protocol was approved by the University of Groningen Ethical Committee of the Psychology Department.

## Author Contributions

CS, ME, CB, and MN designed the study; CS acquired and analyzed the data; CS and ME wrote the first draft of the article; CS, ME, CB, EH, and MN contributed to the interpretation of the results and the writing of the manuscript. All authors have approved the final manuscript.

## Conflict of Interest Statement

The authors declare that the research was conducted in the absence of any commercial or financial relationships that could be construed as a potential conflict of interest. EH serves on the Board of the Charity “MQ; transforming mental health,” and is chair of the fellows committee, and has received no remuneration for this role. EH is an Honorary Professor of Clinical Psychology at the University of Oxford, Department of Psychiatry, and receives no remuneration for this role. EH is Official Fellow at Clare Hall Cambridge, UK and receives a food allowance for this role. EH is on the Board of Overseers for the charity “Children and War Foundation,” Oslo, Norway and receives no remuneration for this role. EH is Associate Editor of “Behavior Research and Therapy” and receives honorariums for this role. EH is on the editorial board of “Cognitive Behavior Therapy” and “Psychological Science” and she receives no remuneration for these roles. EH has received the Humboldt Foundation Friedrich Wilhelm Bessel Research Award (Germany), 2013; and the American Psychological Association (APA) Distinguished Scientific Award for Early Career Contribution to Psychology in the area of Psychopathology, 2014, and these are not directly related to the current Contribution. EH receives travels expenses, some subsistence, and associated speaker honorarium for keynote at conferences, e.g., EABCT 2016. EH presents at clinical training workshops, some of which include a fee, e.g., Copenhagen; Iceland. EH receives royalties from her co-authored book on Imagery in Cognitive Therapy (OUP, 2011). CB is Professor of Clinical Psychology at the Department of Clinical Psychology at the University of Utrecht in The Netherlands (primary affiliation) and University of Groningen. She is also a Guest Professorship at the Faculty of Psychology and Pedagogy at Ghent University in Belgium. CB serves on the board of the section Affective disorders at Dutch Cognitive Behavioral Association and is member of the Dutch multi-disciplinary guideline for anxiety and depression. She receives no remuneration for this role. CB is coeditor of “PlosOne” and receives no honorarium for this role. She is advisor for the National Health Care on forms of care for inclusion in the statutory insured package (Advies Pakket Commissie, ZIN). She receives honorarium for this role and this role has no direct relation to the current Contribution. CB has received the NIAS theme group fellowship 2017 supported by Royal Netherlands Academy of Arts and Sciences (KNAW), and this is not directly related to the current Contribution. CB has presented keynote addresses at conferences such as EABCT 2014 and received an honorarium. She has presented clinical training workshops, some of which include a fee. CB receives royalties from her books and coedited books. MN is a member of the Dutch Association of Behavioral and Cognitive Therapies and receives no remuneration for this role. She developed CBT treatment manuals, including a blended Internet-based treatment program, for which she receives no personal fees. MN is a member of the task force of the Dutch National Care Standard for anxiety disorders (Zorgstandaard Angststoornissen), for which she received travel expenses and some subsistence. MN receives travels expenses, some subsistence, and sometimes an associated speaker honorarium for lectures or clinical training workshops. MN has received grants from ZOnMW (The Netherlands Organisation for Health Research and Development) are unrelated to the current topic of interest.
