# Supplementary material for: Rethinking a Negative Event: The Affective Impact of Ruminative versus Imagery-Based Processing of Aversive Autobiographical Memories
Source: Front Psychiatry. 2017 May 30;8:82. doi: 10.3389/fpsyt.2017.00082 (PMC5447674; doi:10.3389/fpsyt.2017.00082)
Supplement: Supplementary file 1 [file Table_1.DOCX]

Supplementary Material

Rethinking a Negative Event: The Affective Impact of Ruminative versus Imagery-Based Processing of Aversive Autobiographical Memories

Christien Slofstra^1^, Maarten C. Eisma^1^, Emily A. Holmes^3^, Claudi L. H. Bockting^1,2*^, Maaike H. Nauta^1^

*** Correspondence:** Maaike H. Nauta: m.h.nauta@rug.nl

# Supplementary Table

Table S1

State processing mode measures. “When you just recalled the memory, to what degree did you…”. Means and SDs of first recall (pre-manipulation; spontaneous recall).

|  | Mean (SD) |
| --- | --- |
| **Abstract verbal** | **42 (25)** |
| *…*think about the meaning of the event | 51 (32) |
| *…*analyze the meaning of your feelings | 40 (32) |
| …think about why you reacted the way you did | 33 (34) |
| **Concrete verbal** | **50 (30)** |
| …think in words | 48 (33) |
| …formulate your thoughts in sentences | 52 (33) |
| **Imagery** | **43 (19)** |
| …see the memory in your mind’s eye | 78 (25) |
| …hear the memory in your mind’s ear | 41 (35) |
| …smell the fragrances associated with the memory | 9 (18) |
| …feel the physical sensations accompanying the memory | 45 (35) |
